# Advancements in Agricultural Nanotechnology: An Updated Review

**DOI:** 10.3390/plants14182939

**Published:** 2025-09-22

**Authors:** Mario Pagano, Erika Lunetta, Francesco Belli, Giacomo Mocarli, Claudia Cocozza, Ilaria Cacciotti

**Affiliations:** 1 Institute of Research on Terrestrial Ecosystems (IRET), National Research Council (CNR), Via Madonna del Piano 10, 50019 Florence, Italy; 2 Engineering Department, INSTM RU, University of Rome Niccolò Cusano, Via Don Carlo Gnocchi 3, 00166 Rome, Italy; erika.lunetta@unicusano.it (E.L.); ilaria.cacciotti@unicusano.it (I.C.); 3 Department of Agriculture, Food, Environment and Forestry (DAGRI), University of Florence, Piazzale delle Cascine 18, 50144 Florence, Italy; francesco.belli2@edu.unifi.it (F.B.); giacomo.mocarli@edu.unifi.it (G.M.); claudia.cocozza@unifi.it (C.C.)

**Keywords:** agricultural nanotechnology, nanofertilizers, nanopesticides, nanocarriers, nanosensors, sustainable agriculture, environmental sustainability

## Abstract

Sustainable agriculture aims to meet the growing food demands of a rising global population while minimizing negative impacts on the environment, preserving natural resources, and ensuring long-term agricultural productivity. However, conventional agricultural practices often involve excessive use of chemical fertilizers, pesticides, and water, leading to soil degradation, water pollution, and ecosystem imbalances. In this context, agricultural nanotechnology has emerged as a transformative field, offering innovative solutions to enhance crop productivity, improve soil health, and ensure sustainable agricultural practices. This review has explored the wide-ranging uses of nanotechnology in agriculture, highlighting innovative plant-targeted delivery systems—such as polymer-based nanoparticles, carbon nanomaterials, dendrimers, metal oxide particles, and nanoemulsions—as well as its contributions to minimizing pesticide application, alleviating plant stress, and improving interactions between plants and nanoparticles. By examining recent research and development, the review highlights the potential of nanotechnology to address critical challenges such as pest resistance, nutrient management, and environmental sustainability. In conclusion, we believe that, in the immediate future, key priorities should include: (1) scaling up field trials to validate laboratory findings, (2) developing biodegradable nanomaterials to ensure environmental safety, and (3) integrating nanotechnology with digital agriculture platforms to enable real-time monitoring and adaptive management. These steps are essential for translating promising research into practical, sustainable solutions that can effectively support global food security.

## 1. Introduction

**Figure 1:**
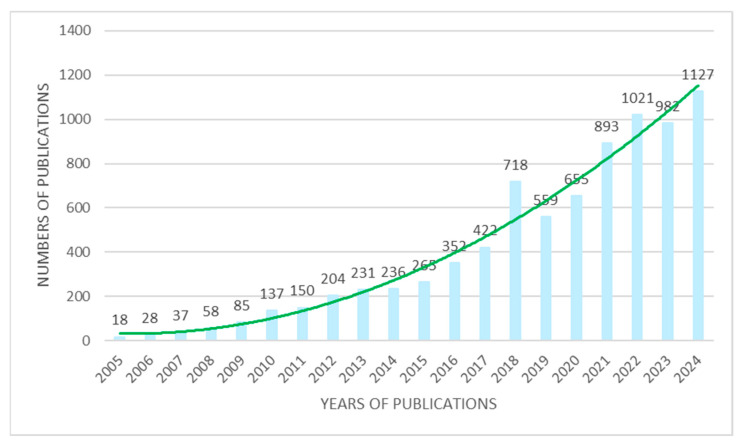
A total of 8178 publications related to the terms ‘nanotechnology and agriculture’ from 2005 to 2024 have been indexed in the Web of Science. The growing interest in this topic is clearly illustrated by the second-degree polynomial trendline shown in green.

Agriculture, which encompasses the diverse methods by which cultivated plants and domesticated animals support the global population through the provision of food and other essential goods [1], has served as a fundamental pillar of human civilization, delivering nourishment, economic value, and critical resources throughout history. As we move further into the 21st century, its role has become increasingly critical. With the global population rising and environmental issues intensifying, agriculture must now tackle the pressing challenges of food security, sustainability, and climate resilience [2,3]. According to projections by the United Nations, the world population is expected to reach 9.7 billion by 2050. Meeting the demands of this growing population will require a 70% increase in food production. This significant demographic shift places considerable strain on the agricultural sector to boost output and improve overall efficiency [4]. In this evolving landscape, the integration of nanotechnology into agriculture presents exciting possibilities, offering cutting-edge solutions to global problems and transforming the future of farming [2,3]. The word “nano” means one-billionth of a meter, so nanotechnology works with materials that are extremely small. What makes nanotechnology special is that it allows scientists to work at the level of molecules and atoms to build complex structures with new and unique organization [5,6]. Agri-nanotechnology, which integrates nanoscience with agricultural practices, is an emerging field of research and innovation with significant potential to promote sustainable farming methods and enhance global food security [7,8]. Agricultural nanotechnology involves the application of nanoproducts and nanotechnologies across various areas of the agricultural sector, such as enhancing crop yields, improving soil quality, and promoting sustainable farming practices. This technological approach offers a broad spectrum of applications, including nanosensors for monitoring plant health and nanofertilizers for efficient nutrient delivery [9]. Another important aspect is crop protection, which can be significantly enhanced using nanomaterials such as nanoparticles and nanocomposites (with dimensions ranging from 1 to 100 nanometers [10]) [11]. Specifically, nano-enabled agrochemicals offer increased efficacy, targeted delivery, and controlled release, leading to more effective pest and disease management. This approach not only improves crop health but also helps minimize the environmental risks associated with traditional agrochemicals [7,12]. In addition, nanoscale delivery systems play a key role in biofortification by addressing nutritional deficiencies. Through nanoencapsulation, essential nutrients can be released in a controlled manner, enhancing their uptake by plants and ultimately improving the nutritional quality of crops [13]. The relevance of the topic presented is clearly illustrated in Figure 1, where the second-degree polynomial trendline, shown in green, highlights the increasing number of publications related to the terms ‘nanotechnology and agriculture’ from 2005 to 2024, as indexed in the Web of Science database. Through an analysis of recent advancements in research and development, this review underscores how nanotechnology can contribute to solving key issues such as pest resistance, efficient nutrient use, and promoting environmental sustainability. This review provides an updated perspective compared in agricultural nanotechnology to previous publications. It also includes a more current and comprehensive set of references. In particular, while it is true that nanotechnology in agriculture has been widely studied, this review contributes to the field by offering a comprehensive and integrative perspective on the most recent advancements, with a particular focus on plant-targeted delivery systems and their role in promoting sustainable agricultural practices. Specifically, the article adds value in the following ways: Synthesis of Emerging Nanocarriers: it highlights and compares novel nanocarriers—such as polymer-based nanoparticles, carbon nanomaterials, dendrimers, metal oxide particles, and nanoemulsions—emphasizing their unique properties and mechanisms for targeted delivery in plants. Focus on Sustainability: unlike many previous studies that focus primarily on efficacy, this review frames nanotechnology within the broader context of environmental sustainability, addressing how these interventions can reduce chemical inputs, mitigate plant stress, and improve soil health. Bridging Knowledge Gaps: the review identifies critical challenges such as pest resistance and nutrient inefficiency and discusses how nanotechnology can offer multifunctional solutions to these issues, thereby contributing to long-term agricultural resilience. Recent Research Integration: by incorporating up-to-date findings, the article provides a timely overview of current trends and innovations, helping researchers and practitioners understand the trajectory of the field and identify promising areas for future investigation. In summary, this review does not merely reiterate existing knowledge but rather reframes and updates it through the lens of sustainability and innovation, offering a strategic roadmap for future research and application in agricultural nanotechnology.

## 2. From Interaction to Innovation: Nanoparticles in Plant Science and Sustainable Agriculture

**Figure 2:**
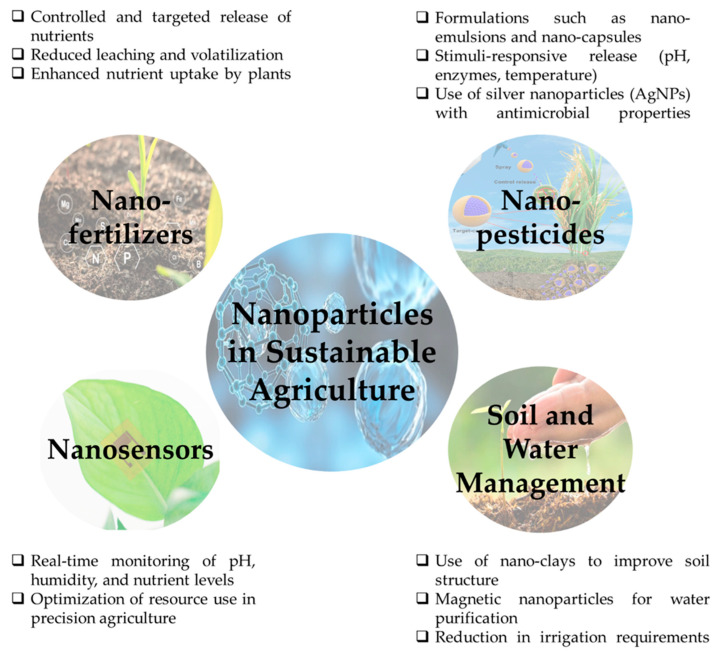
Different applications of nanoparticles in sustainable agriculture.

Among various applications of nanotechnology, the use of nanoparticles (NPs) stands out due to their unique physicochemical properties, such as high surface area, reactivity, and the ability to interact with biological systems at the molecular level [14]. Nanoparticles can be used for different applications. They include nanoformulated herbicides (e.g., atrazine-loaded PCL nanoparticles) showing enhanced weed control with less impact on crops [15]; fungicide-loaded nanoparticles (e.g., chitosan-based) that increase antifungal activity and reduce phytotoxicity [16]; insecticide nanoparticles that improve efficacy and reduce toxicity to non-target species [17]; and nanoparticle delivery of plant growth regulators to enhance plant stress tolerance and growth [18]. The key applications of nanoparticles in sustainable agriculture, including nano-fertilizers, nano-pesticides, nanosensors, and their roles in soil and water management are summarized in Figure 2 and Table 1.

## 3. Nanotechnology in Plant Drug Delivery

**Figure 3:**
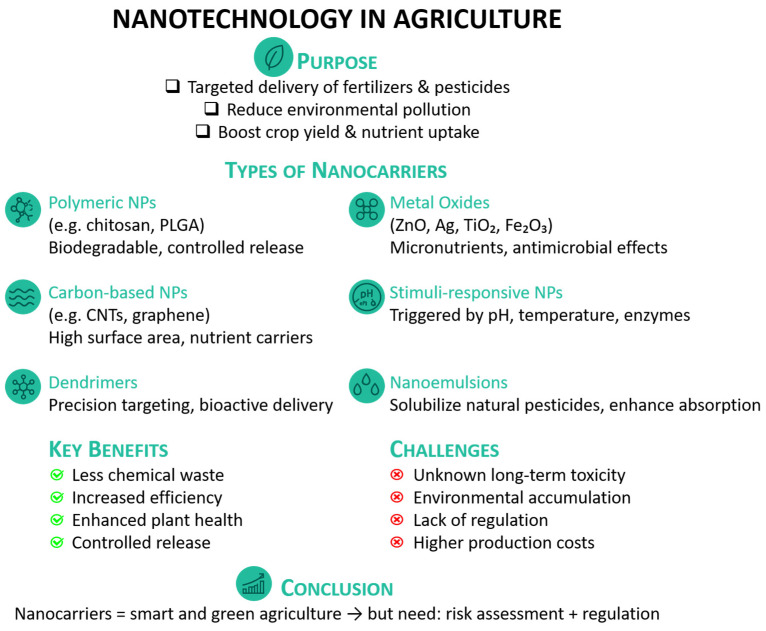
Overview of nanocarriers used in environmental remediation, highlighting their types, key benefits, and associated challenges.

Nanotechnology is playing an innovative role in agriculture by enabling precision delivery of agrochemicals to plants. This approach not only improves the efficiency of active compound usage but also minimizes environmental impact, waste, and off-target toxicity [31]. Nanocarriers have emerged as a fundamental tool in modern agriculture, offering a smart and targeted approach to delivering bioactive compounds to plants [32]. These nanoscale systems are typically engineered from a variety of materials, including biodegradable polymers (e.g., chitosan, poly(lactic-co-glycolic) acid—PLGA), lipids, silica, or metallic nanoparticles, each tailored to optimize stability, compatibility, and release behavior [33]. By encapsulating fertilizers, pesticides, herbicides, or plant growth regulators, nanocarriers enable controlled and site-specific release, often triggered by environmental conditions such as pH, temperature, or enzymatic activity. This precision delivery minimizes off-target effects, reduces the employment of chemicals, and enhances the efficacy of treatments. In agronomic contexts, nanocarriers show promise in improving crop yield, nutrient uptake, and resistance to pests and diseases, paving the way for more sustainable and efficient farming practices than the traditional ones [34]. Figure 3 summarizes the purpose, various types, key benefits, and major challenges of using nanocarriers—such as polymeric nanoparticles, dendrimers, and nanoemulsions—for environmental remediation applications.

The application of nanoparticles in agriculture holds great promise for improving nutrient delivery, pest control, and stress tolerance. However, several challenges must be addressed for their safe and effective use. One major challenge is phytotoxicity: NPs can be harmful to plants at high concentrations, causing oxidative stress, membrane damage, or growth inhibition [35]. The effects depend on the type, size, and shape of the nanoparticles, as well as the plant species, making precise dosing difficult. Environmental concerns are another issue, as NPs may accumulate in soil or water, potentially affecting non-target organisms and disrupting ecosystems. Limited targeting and uncontrolled release of nutrients or pesticides can reduce efficiency and increase environmental contamination. Regulatory and safety issues also slow commercialization, as standardized testing protocols and clear guidelines for food crops are still lacking [36]. Finally, the cost and scalability of nanoparticle production, along with storage and stability issues, present additional hurdles. Modern biotechnologies provide several strategies to overcome these challenges [37]. Biopolymeric nanoparticles made from biodegradable polymers, such as chitosan, alginate, or PLGA, can reduce phytotoxicity and environmental accumulation while enabling controlled release of nutrients and bioactive compounds. Stimuli-responsive nanocarriers, designed to release their contents in response to pH, light, temperature, or enzymatic activity, allow precise delivery and minimize off-target effects. Genetic and metabolic engineering of crops can enhance nanoparticle uptake or tolerance by improving efficiency and reducing toxicity. Finally, eco-friendly synthesis methods using plant extracts or microbes produce biocompatible nanoparticles with lower environmental risk, increasing public acceptance [38].

Table 2 categorizes the most researched and applied nanocarriers in agriculture, outlining their base materials, mechanisms of action, and agronomic applications. Nanoparticles used in agriculture can be classified according to their composition into several categories, including metal and metal oxide nanoparticles (e.g., zinc oxide, silver, iron oxide), carbon-based nanoparticles (e.g., carbon nanotubes, fullerenes), and polymeric nanoparticles, as described in the following paragraphs in detail.

### 3.1. Biopolymeric Nanoparticles

Polymeric nanoparticles can be synthesized using chemical, physical, or biological methods [58]. Green synthesis using plant extracts or microbes is gaining attention due to its environmental friendliness [59,60]. The nanoscale size of these particles allows for enhanced solubility, controlled release, and targeted delivery of active ingredients [61]. Moreover, their large surface-to-volume ratio facilitates and improves the interaction with plant cells and soil particles. These properties make nanoparticles particularly suitable for precision agriculture, where inputs are optimized both spatially and temporally [62]. In more detail, biopolymeric nanoparticles are nanoscale particles formulated using biocompatible polymers, either natural (e.g., chitosan, alginate) or synthetic (e.g., PLGA, poly(ε-caprolactone)—PCL, polyglutamic acid or polyglycolides—PGA, poly(vinyl alcohol)—PVA, poly(citric acid)—PCA, polyethylene glycol—PEG) (Table 2). They offer excellent stability, biodegradability, and can be engineered to degrade over time, allowing for a sustained release of encapsulated pesticides, fertilizers, or plant hormones [21]. Despite promising laboratory results, challenges remain for large-scale production, toxicity evaluation, environmental impact assessment, and consumer acceptance [21].

For instance, chitosan NPs (1000–5000 ppm) reduced Fusarium infection in wheat by 40–60% while showing lower phytotoxicity compared to bulk chitosan. Alginate/chitosan nanoparticles were developed as carriers for the hydrophobic compound quercetin, a polyphenolic flavonoid with multiple bioactive properties by Nalini et al. The NPs were prepared via ionotropic gelation of chitosan with sodium tripolyphosphate, followed by alginate polyelectrolyte complexation. Characterization by ATR-FTIR, SEM, TEM, and XRD confirmed their well-defined spherical architecture with particle sizes ranging from 118 to 255 nm. They exhibited high quercetin encapsulation efficiency (82.4%) and loading capacity (46.5%), demonstrating their potential as effective and stable delivery systems for bioactive compounds in plants [63]. The study of Badiali et al. investigated the effects of free pterostilbene (PTB) and PLGA NPs loaded with PTB on the metabolism of tomato (*Solanum lycopersicum* L.) leaves. Using untargeted 1H-NMR metabolomics and targeted HPLC–MS/MS lipidomics, they found that both PTB and PLGA NPs + PTB increased γ-Aminobutyric acid (GABA) levels, shifted secondary metabolism toward phenylpropanoid biosynthesis, and modulated fatty acids and oxylipins. The treatments also influenced hormones involved in plant defense, such as salicylic and jasmonic acid, suggesting a redirection of leaf metabolome to enhance antioxidant and stress-response pathways. These results indicate that PLGA NPs loaded with PTB could serve as priming agents to improve plant resilience and production of bioactive compounds, with minimal metabolic disruption. Further studies are needed to optimize concentrations, application methods, and developmental timing for crop improvement under environmental stress [64]. The study of Olad et al. reports the synthesis of a novel slow-release nitrogen-phosphorus-potassium (NPK) fertilizer hydrogel nanocomposite (Hydrogel/Polyvinylpyrrolidone/Silica/NPK) via in situ graft polymerization of acrylate-based monomers onto a sulfonated-carboxymethyl cellulose (SCMC) backbone in the presence of polyvinylpyrrolidone (PVP), silica nanoparticles, and NPK fertilizer. FT-IR and XRD analyses confirmed successful grafting of SCMC with acrylate monomers. The hydrogel demonstrated good pH and salt sensitivity, excellent water retention in loamy sand soil, and slow-release behavior of NPK following a Fickian diffusion mechanism. These properties suggest that the nanocomposite can enhance fertilizer efficiency and support water conservation in agricultural and horticultural applications [65]. The study by Al-Surhanee et al. evaluated the in vitro and greenhouse antifungal activity of silver nanoparticles (Ag NPs), chitosan nanoparticles (CHI NPs), and their composites (Ag NPs/CHI NPs) against Rhizoctonia solani, a fungus harmful to tomato crops. Plant health was assessed using transmission electron microscopy (TEM) and FTIR spectrophotometry. The results showed a significant improvement in the health of plants treated with the Ag/CHI NPs composites, suggesting their potential use at both experimental and commercial levels to manage *R. solani* pathogenicity and enhance tomato cultivation [66].

Generally, biopolymeric nanoparticles represent a promising technology to increase agricultural productivity sustainably by maximizing effectiveness while minimizing environmental risks.

### 3.2. Carbon-Based Nanoparticles

Carbon-based nanoparticles, such as carbon nanotubes (CNTs) and fullerenes, are increasingly being explored as efficient nanocarriers in agriculture. Their unique structural properties, including high surface area, chemical stability, and the ability to penetrate biological membranes, make them suitable for transporting agrochemicals. When used as delivery systems, these nanoparticles can improve the solubility and stability of active compounds, allowing for more precise and sustained release at target sites within plants or soil (Table 2). This targeted delivery not only enhances the effectiveness of the applied substances but also helps reduce overall usage and environmental impact [67]. Additionally, surface functionalization of carbon nanoparticles enables them to selectively interact with plant tissues, further improving uptake and minimizing off-target effects. For example, CNTs have been shown to cross cell walls via root or foliar application, acting as slow-release vehicles that enhance nutrient uptake (nitrogen, phosphorus, potassium) and boost crop yield while limiting environmental runoff [39]. Graphene oxide (GO) sheets can carry trace elements like zinc and copper, releasing them in stages (an initial burst followed by sustained delivery) leading to improved absorption in wheat [41]. CNTs grafted with functional polymers can also serve as carriers for antifungal agents, showing enhanced stability and efficacy versus conventional formulations. Biochar-derived nanoparticles further demonstrate promise: rice husk biochar NPs loaded with herbicides reduce leaching, prolong soil retention, and minimize groundwater contamination [68]. Moreover, core–shell nanocarbon composites, composed of nanocarbon as the core, amino-functionalized mesoporous silica (mSiO_2_-NH_2_) as the shell, and polydopamine (PDA) as the coating layer, are used as foliar fertilizers. They have achieved yield increases nearing 70%, improved nitrogen use efficiency, and reduced nutrient losses [40]. Carbon nanotubes, when applied at concentrations of 20–100 mg/L, significantly enhanced nutrient uptake (N, P, K) and increased biomass production in maize compared to conventional fertilization strategies. Their high surface area and ability to penetrate plant tissues facilitate efficient nutrient transport and assimilation. Moreover, CNTs have been reported to stimulate root growth and improve water use efficiency, suggesting their potential as nanoscale carriers to boost crop productivity in a more sustainable way [41].

Despite their potential, further studies are needed to fully understand their long-term environmental safety and impact on plant health.

### 3.3. Dendrimers

With respect to dendrimers, they are synthetic, tree-like branched nanostructures with numerous surface groups that can be modified for targeted binding [22]. They have precise molecular weights, large internal cavities, and high functional surface density, making them ideal for multi-functional delivery [69]. Although the use of dendrimers as drug delivery systems is well established in the medical field, their application in agriculture is still under development. However, the principles and techniques employed in the medical sector could be adapted to enhance the effectiveness of pesticides and fertilizers through controlled and targeted release [70]. One of the key advantages of dendrimers is their ability to carry bioactive phytochemicals, natural compounds with pesticidal or growth-promoting properties, that often suffer from poor solubility and instability (Table 2). By encapsulating these molecules, dendrimers can enhance their solubility, protect them from degradation, and provide sustained release over time. This not only improves the effectiveness of plant-based pesticides but also reduces the frequency and quantity of chemical applications, making agricultural practices more efficient and environmentally friendly [42]. For instance, poly(amidoamine) (PAMAM) dendrimers have shown potential in encapsulating and delivering natural compounds such as essential oils and plant-based antimicrobials, demonstrating their suitability for agricultural nanocarrier applications [71]. Bunderson et al. realized PAMAM dendrimers of generations 2–3.5 at very low concentrations (1–10 ppb) combined with 12-0-0 fertilizer enriched with iron and manganese. They improved fertilizer mobility and plant growth in foliar applications. MSNs of 20 nm with 2 nm pores were taken up by wheat, lupin, and Arabidopsis without phytotoxicity, localizing in root cells, xylem, and leaf intercellular spaces, highlighting their potential as small-molecule delivery systems [43].

In summary, adapting dendrimer-based delivery strategies from medicine to agriculture offers a promising route to develop smart, eco-friendly crop protection and enhancement systems.

### 3.4. Metal Oxide Nanoparticles

Among metal oxide nanoparticles, mesoporous silica nanoparticles (MSNs) are emerging as promising nanocarriers for the delivery of biomolecules in plants (Table 2). Thanks to their high surface area, tunable pore size, and excellent biocompatibility, MSNs can be engineered to encapsulate and protect sensitive bioactives such as nucleic acids, proteins, and small molecules, ensuring their stability and controlled release [45]. For instance, Hussain et al. explore the MSN synthesis via sol–gel methods and highlight their ability to enhance the efficiency of biomolecule delivery through targeted and sustained release. MSNs have demonstrated non-toxic behavior in plant cells and show potential to overcome traditional delivery challenges, like low penetration and rapid degradation of bioactive compounds. These features make MSNs a valuable material for plant genetic engineering, disease resistance, and crop enhancement [44]. Metal/metal oxide nanoparticles also include particles of zinc oxide (ZnO), iron oxide (Fe_2_O_3_), titanium dioxide (TiO_2_), and silver (Ag NPs) (Table 2), which serve both as micronutrients and antimicrobial agents [49]. In particular, silver nanoparticles are widely recognized for their strong antimicrobial effects, effectively inhibiting fungi, bacteria, and even nematodes. Besides protecting plants from pathogens, Ag NPs have been found to enhance seed germination, stimulate plant growth, and improve photosynthetic efficiency. However, their benefits come with a caution: at very high concentrations or extremely small sizes, silver nanoparticles can become toxic to plant cells, as observed in rice roots [72,73]. Another important delivery is represented by zinc oxide nanoparticles (ZnO NPs), which play a vital role in plant nutrition since zinc is an essential micronutrient involved in enzyme activity and photosynthesis. Application of ZnO NPs to crops such as sesame, peanuts, tobacco, mustard, and fenugreek has led to improved seed germination, enhanced root and shoot growth, increased chlorophyll content, and greater biomass production. Their positive effects heavily depend on the dosage, plant species, and nanoparticle size, as excessive amounts can cause toxicity [74]. Copper nanoparticles (Cu, CuO, Cu_2_O NPs) are mainly appreciated for their antimicrobial properties, particularly against pathogens like *Bacillus subtilis*. Although copper nanoparticles also find use in catalytic and electronic fields, their agricultural relevance primarily lies in disease control, helping reduce infections and improving crop health [75]. For instance, SiO_2_ NPs alleviated CuO NP-induced root growth inhibition in sorghum, wheat, and rye at 100–800 mg/L, whereas in triticale the effect was reversed. Metal/metal oxide nanoparticles, such as Fe_3_O_4_ at 200–500 mg/L, enhanced wheat growth, photosynthesis, respiration, leaf pigment and nutrient content, and antioxidant activity [47].

Iron oxide nanoparticles (Fe_3_O_4_ and Fe_2_O_3_ NPs) are notable for their low toxicity and versatility in biomedical and agricultural applications. Serving as an important iron source, these nanoparticles help fight iron deficiencies in plants by enhancing phytohormone levels, antioxidant activities, and overall plant vitality, as shown in peanut plants [76,77]. Titanium dioxide nanoparticles (TiO_2_ NPs), while classified as non-essential elements for plants, still demonstrate beneficial effects on seed germination and nutrient absorption. Their impact varies with the dose administered and the specific crop, indicating a need for careful optimization [78]. Though less explored in agriculture, gold nanoparticles (Au NPs) are primarily valued for their optical properties and applications in coatings and diagnostics. Recent studies suggest that they also possess antimicrobial activity, for example, against *Escherichia coli*, and may influence plant metabolic processes [79]. Magnesium oxide nanoparticles (MgO NPs) show promising antioxidant and antimicrobial properties combined with low toxicity, which can help protect plants and potentially increase crop yields [80]. Calcium carbonate nanoparticles (CaCO_3_ NPs) are characterized by affordability, biocompatibility, and the ability to release nutrients in a controlled manner. These features make them excellent candidates for use as smart fertilizer carriers, where their pH sensitivity allows for timed and targeted nutrient delivery [81]. For instance, Hua et al. demonstrated that CaCO_3_ NPs sprayed on the leaves of *Citrus tankan* enhanced calcium accumulation and increased tolerance to pests such as the California red scale and *Bactrocera dorsalis*, showing lower 50% lethal concentration (LC50) values compared to colloidal CaCO_3_ [82].

### 3.5. Stimuli-Responsive Nanocarriers in Agriculture

Stimuli-responsive nanocarriers are advanced delivery systems designed to release fertilizers or pesticides in response to specific environmental triggers or stimuli (Table 1). These triggers can be changes in pH, temperature, moisture, redox conditions, light, or enzymes present in the soil or plant environment. This smart release mechanism ensures that agrochemicals are more precisely and efficiently delivered, reducing waste and environmental contamination [25]. For example, pH-responsive nanocarriers release their load when the soil acidity or alkalinity changes, which can happen due to root exudates or microbial activity. This feature allows nutrients or pesticides to be released exactly when plants need them or when pathogens are active [83]. Temperature-sensitive nanocarriers adjust the release rate based on ambient temperature variations. This is useful in climates where pest activity or plant nutrient demands fluctuate with temperature changes, ensuring timely delivery [84]. Redox-responsive nanocarriers respond to the oxidation-reduction conditions found in the rhizosphere (root zone). Such nanocarriers can release pesticides or fertilizers when oxidative stress in plants is detected, offering targeted protection [85]. Another type of stimuli-responsive nanocarriers is the enzyme-responsive one, which degrades in the presence of specific enzymes secreted by soil microbes or plants, triggering the release of their active substances. The employed materials for these nanocarriers include polymers that degrade or swell in response to stimuli, liposomes that change their structure, and inorganic nanoparticles engineered to respond to environmental signals [86]. Stimuli-responsive nanocarriers, such as pectin-coated dendritic mesoporous silica nanoparticles, tested at 50–500 mg/L in tomato, promoted root growth at low concentrations, did not affect seed germination, were safe for non-target organisms, and showed antibacterial activity with EC_50_ values of 126 mg/L for *Bacillus subtilis* and 165 mg/L for *E. coli* [55].

Overall, stimuli-responsive nanocarriers represent a promising approach to improve the precision, efficiency, and sustainability of agrochemical application by ensuring that active compounds are released only when and where they are most needed.

### 3.6. Nanoemulsions

Nanoemulsions are fine dispersions of oil and water stabilized by surfactants, with droplet sizes typically between 20 and 200 nm [87]. Nanoemulsions offer several important advantages when used as a method for delivering active substances in agriculture (Table 1). Due to their extremely small particle size, nanoemulsions significantly improve the solubility and stability of hydrophobic compounds, which enhances the bioavailability of these active ingredients. Their small size also allows the substances to more deeply penetrate into plant tissues, including the cuticle layer, resulting in more effective pest control and nutrient delivery [56]. Furthermore, nanoemulsions can be designed to release their active ingredients in a controlled and sustained manner. This controlled release reduces the frequency of needed application, which not only makes agricultural practices more efficient but also minimizes environmental contamination. By increasing the efficiency of how active ingredients are delivered, nanoemulsions also help reduce the total amount of chemicals required. This reduction consequently lowers chemical runoff and decreases toxicity to non-target organisms, thereby promoting greater environmental sustainability [88]. Despite these advantages, there are some challenges and drawbacks associated with the use of nanoemulsions in agriculture. The very small size and high surface area that make nanoemulsions effective also raise concerns about their potential toxicity to plants, soil organisms, and aquatic life. Additionally, there is a risk of bioaccumulation, where nanoparticles may persist in the environment and enter the food chain, potentially causing long-term ecological effects [89]. Another important issue is the regulatory and safety framework surrounding nanoemulsions. Because this technology is relatively new in the agricultural sector, comprehensive safety assessments are still ongoing, and standardized regulations and guidelines are lacking. This situation complicates the widespread adoption of nanoemulsions and may slow their acceptance by both farmers and consumers. Finally, the production costs for nanoemulsions tend to be higher than those for traditional formulations. This increased cost can limit accessibility, particularly for smallholder farmers, potentially exacerbating inequalities in agricultural practices and benefits [56]. In the study of Rys et al., nanoemulsions of caraway oil applied to leaves up to 10% were harmless to maize but caused 50% and 90% damage in barnyard grass at 5.1% and 13% concentrations, respectively, reducing leaf relative water content and demonstrating selective herbicidal effects [57].

In summary, while nanoemulsions represent a promising advancement for agricultural drug delivery by improving efficacy and sustainability, their successful implementation depends on addressing environmental safety concerns, developing clear regulatory frameworks, and ensuring economic accessibility for all farmers.

## 4. Nanotechnology for Efficient Use of Fertilizers

Among the major challenges in agriculture, the inefficient use of fertilizers has attracted a lot of attention: traditional fertilizers often suffer from low nutrient-use efficiency, with significant losses due to leaching, volatilization, or fixation in the soil. Thus, to overcome this criticism, the development of nano-fertilizers, materials that contain nutrients either encapsulated within nanoparticles or coated onto their surface, has been proposed [25]. Nano-fertilizers can release nutrients in a controlled and sustained manner, matching the crop’s growth cycle and nutrient requirements. For instance, ZnO NPs have been shown to enhance zinc uptake in crops, improving growth and yield while using lower doses than conventional fertilizers [24]. Similarly, nano-encapsulated nitrogen fertilizers limit nitrogen losses and increase nitrogen-use efficiency, thereby reducing the environmental impact associated with nitrogen leaching and greenhouse gas emissions [24]. Moreover, the enhanced adhesion and penetration capabilities of nanoparticles enable better leaf coverage and uptake, leading to increased pesticide efficiency and reduced environmental contamination [22].

## 5. Nanotechnology for Efficient Water Use and Soil Health Maintenance

**Figure 4:**
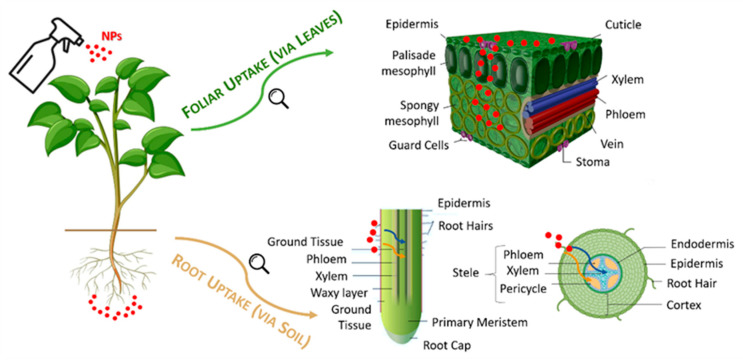
Summary of nanoparticle uptake mechanisms in plants (elaborated from [94]).

Efficient water use and soil health maintenance are critical components of sustainable agriculture. Nanoparticles play a role in both areas. For example, nano-clays and other nanomaterials can improve soil structure, enhance water retention, and aid in slow-release fertilizer formulations [27]. In drought-prone areas, such improvements can significantly reduce irrigation requirements. Nanoparticles are also being employed for water purification in agricultural settings. Magnetic nanoparticles, for example, can remove heavy metals and pesticide residues from water sources, providing safer irrigation water and reducing contamination risks [28]. Additionally, nano-enabled sensors are being developed to monitor soil moisture and nutrient levels in real-time, supporting data-driven and precise agricultural interventions [29]. Several studies have demonstrated the positive effects of nanoparticles on plant growth and development [50,90,91]. By enhancing photosynthesis, nutrient uptake, and enzyme activity, nanoparticles can improve plant vigor and productivity. Iron oxide nanoparticles, for instance, have been reported to boost chlorophyll content and photosynthetic efficiency [51]. In crop bioengineering, the applications of NP-mediated transient systems serve as an excellent platform for conducting functional genomic studies in wheat, including the validation of gene functions, protein interactions and regulation, omics studies, and genome editing [30]. Figure 4 summarizes the pathways and mechanisms by which nanoparticles can enter plants. Moreover, the NPs uptake has been recently studied on tree species, by observing that NPs enter the tree stem through leaves faster than through roots [92] and by showing that the surface charge of NPs may play a role in the adhesion and uptake, but not in the transport to tree compartments [93].

## 6. Nanotechnology for Reducing Pesticide Use

Pesticides are chemical substances or mixtures of biological agents used on purpose to prevent, control, or kill harmful organisms (insects, weeds, rodents, fungi, and other pests). They work as destroying, repelling, or mitigating agents; depending on their target, they are called herbicides, insecticides, fungicides, rodenticides, molluscicides, nematicides, or plant growth regulators [95,96]. Pesticide overuse poses significant risks to human health and biodiversity. Their use has grown drastically, reaching about 5.2 billion pounds a year, and has helped cut food losses from pests by up to 45% [95,97]. However, it has also caused serious health and environmental problems: short-term exposure can lead to headaches, skin and eye irritation, stomach issues, dizziness, and in extreme cases blindness or death, while long-term exposure is linked to immune system damage, nerve damage, hormone disruption, and higher cancer rates among farm workers [95,98,99]. Many pesticide products contain not only active ingredients but also “inerts” (solvents, surfactants, preservatives) and process contaminants (like dioxins), which add to their overall toxicity [100]. Widespread use has polluted air, soil, and water contributing about 6% to ground-level ozone formation [98,101] and has left persistent chemicals like DDT (dichlorodiphenyltrichloroethane), chlordane, and dieldrin in the soil, with long-term impacts on ecosystems and biodiversity [97]. Children, pregnant women, and older adults are especially at risk, as even low levels can cause significant harm [99].

In recent years, nanotechnology in agriculture gained a lot of global attention because it can help detect, break down, and remove harmful pesticides, through nano-formulated pesticides [101]. Many different types of nanomaterials, such as nanoparticles, nano-emulsions, nano-capsules, nanotubes, and nanocomposites have been widely studied for this purpose, due to their unique features. As evidenced in the previous paragraphs: they are very small, have a large surface area compared to their volume, special physical and chemical properties, and high ability to target specific substances like pesticides [101]. They can improve the solubility, stability, and targeted delivery of active compounds [23]. Nano-formulated agrochemicals can be engineered to release their active ingredients only in response to specific stimuli (such as pH, temperature, or enzymatic activity), minimizing off-target effects and reducing the required dosage. For example, silver nanoparticles have been effectively used to combat plant pathogens, due to their intrinsic antimicrobial properties [53]. There are two main ways nanomaterials are used to clean up pesticides: homogeneous and heterogeneous methods [102]. In the homogeneous method, nanoparticles are mixed directly into water that contains pesticides; then, these nanoparticles find and destroy the pesticides. In the heterogeneous method, the nanoparticles are first attached to solid materials; these supports are then added to the water, and the attached nanoparticles detect and break down the pesticides there [101].

Furthermore, many insecticides have low water solubility and require organic solvents to help solubilize the pesticide, increasing their toxicity and cost. Nanoparticles such as polymers, silica, and chitosan can improve solubility, reduce volatilization, and enhance stability, while allowing a slow, controlled release of the active compounds [103]. Chitosan nanoparticles loaded with salicylic acid have been used to induce systemic resistance in plants against pathogens [19]. Their mucoadhesive and cationic nature also helps them effectively interact with plant surfaces, promoting uptake [20]. Silica nanoparticles can help reduce plant pests, boost plant growth and yields, and serve as effective alternatives to chemical fertilizers thanks to their easy production, high absorption, and large surface area. They can be used by adding them to the soil or spraying them on leaves. They can enter the plant through the cuticle or stomata and move inside the leaves [104]. It was found that, even at low concentrations, silica nanoparticles can boost antioxidant enzymes like catalase and superoxide dismutase. In *Plutella xylostella* (diamondback moth), a major pest of cruciferous crops, led to increased mortality due to damage to the cuticle, blocked spiracles, and dehydration, even if there were no effects on reproduction or growth. A 2013 study conducted by H. M. El-bendary and A. A. El-Helaly from the Faculty of Agriculture at Cairo University investigated the use of hydrophilic nano-silica particles to tomato plants, to assess their resistance to *Spodoptera littoralis* (cotton leafworm). The goal was to contribute useful insights for managing this pest in tomato cultivation. The results suggested that nano-silica application can moderately reduce insect-related damage, offering a potential support tool in integrated pest management strategies [5].

## 7. Mitigating Plant Stress Using Nanomolecular Techniques

Plants can be exposed to various types of stress, which are generally classified into two main categories: biotic and abiotic stress. Biotic stress refers to stress caused by living organisms, such as fungi or bacteria, while abiotic stress is caused by non-living factors, including drought, pollution, or other climatic conditions affecting agriculture worldwide [105].

As previously mentioned, the most common types of abiotic stress include salinity, heavy metals, suboptimal temperatures, waterlogging, and others [105]. Moreover, the effects of these different stress factors are exacerbated by climate change, particularly in relation to temperature fluctuations and waterlogging [105]. One of the main consequences of stress conditions in plants is a reduction in most growth parameters. In recent years, numerous studies have investigated the interactions between nanoparticles and plants, focusing on their potential to alleviate stress conditions. It has been demonstrated that the use of nanoparticles can enhance plant resilience to abiotic stresses such as drought, salinity, and extreme temperatures, providing numerous benefits for crop production, such as increasing yields, reducing nutrient loss, and helping to manage plant diseases [106]. This is partly due to their role in activating antioxidant defense mechanisms in plants. For example, silica nanoparticles (SiO_2_ NPs) help in reducing oxidative stress and improving water-use efficiency, making them valuable in mitigating the effects of climate change on crop production [46]. According to Kovács et al., silica nanoparticles have been shown to increase it by mitigating CuO-NP-induced stress in crops [47]. In detail, SiO_2_ NP treatment regulated reactive oxygen and nitrogen species in response to CuO-NP-induced stress for enhancing resilience in monocots. Moreover, CuO-NPs could be employed in foliar sprays for controlling plant virus infections, as well as in the case of tobacco infected by alfalfa mosaic virus [107]. Nanoparticles of copper oxide (CuO) and zinc oxide (ZnO) decrease the impact of salt stress on the growth and physiology of *Raphanus sativus* by reducing oxidative stress and declining proline, anthocyanin, and flavonoids contents and enzymatic activities such as superoxide dismutase, ascorbate peroxidase and guaiacol peroxidase [54]. Similarly, Jacobson et al. [108] studied how CuO and ZnO nanoparticles can help plants better adapt to drought conditions, for example, by modifying certain morphological parameters or inducing shoot lignification. The application of Cu-NPs in combination with a microalgae extract, i.e., spirulina, is suggested for the best growth and highest oil production from the French basil plant [109]. In detail, the Cu-NPs fertilizer induces a significant increase in the antioxidant activity of the French basil plant, while the combination of Cu-NPs with spirulina extract causes a decrease in antioxidant activity. The use of nanosilicon excited the activation of antioxidant enzymes, by inducing high superoxide dismutase (SOD), peroxidase (POD), and ascorbate peroxidase (APX) activities, and decreasing proline content, as consequence of an improved drought tolerance in hemp plants [48]. NPs can be used to guarantee the cormlet production, as in the case of the supply of *Trichoderma harzianum* and nano-TiO_2_ that produces larger, more vigorous saffron cormlets with enhanced metabolic profiles [26]. This application represents a practical strategy for cultivation under increasing temperature stress conditions in commercial greenhouse production systems. Similarly, Jaberzadeh et al. [110] demonstrated that TiO_2_ nanoparticles, when applied at a concentration of 0.02% (*w*/*v*), can increase gluten and starch content in wheat under water deficit stress. However, the consistently increasing use of nanoparticles (as well as ZnO-NPs) in crop optimization practices and their persistence in the agro-environment necessitate expounding on their influence on sustainable agro-environment. In the context of environmental sustainability, the adsorption of iron oxide nanoparticles reduces the toxicity and bioavailability of nanoplastics in soil [52]. Iron oxide nanoparticles limit the uptake of polystyrene nanoplastics by improving plant biomass, photosynthetic performance, and by mitigating nanoplastics-induced oxidative stress through flavonoid biosynthesis pathway activation.

## 8. Concluding Remarks and Future Perspectives

Agricultural nanotechnology has emerged as a groundbreaking frontier in the pursuit of sustainable and efficient farming practices, to enhance the efficiency and sustainability of agricultural practices. This review has examined the diverse applications of nanotechnology in agriculture, including advanced plant drug delivery systems—such as polymeric nanoparticles, carbon-based nanoparticles, dendrimers, metal oxide nanoparticles, and nanoemulsions—as well as its role in reducing pesticide use, mitigating plant stress, and enhancing plant-nanoparticle interactions.

The integration of nanoparticles into agricultural systems offers promising benefits, from improving nutrient use efficiency and crop resilience to minimizing environmental impact. Metallic nanoparticles (MNPs) show significant potential in agriculture due to their ability to damage pests by interacting with the exoskeleton and penetrating cell membranes, as in the case of AgNPs smaller than 10 nm, and by stimulating immune responses that promote pest elimination [111]. While the benefits of using nanoparticles in agriculture are promising, concerns remain regarding their potential environmental and health impacts.

In fact, despite these advantages, concerns persist regarding the environmental fate, bioaccumulation, and long-term effects of nanoparticles on ecosystems and human health. Addressing these challenges requires the development of biodegradable nanomaterials, robust regulatory frameworks, and transparent risk assessments. Some studies report no significant toxicity of silver nanoparticles, either in vitro or in vivo, even under conditions considered critical in the literature [112]. However, nanoparticles still represent a notable environmental challenge. Their persistence, bioaccumulation potential, and cellular interactions can disrupt soil microbial communities and soil-dwelling organisms. This disruption may negatively affect plant growth and soil fertility [32]. While offering innovative opportunities, NPs pose risks to biodiversity and ecosystems, and their use requires caution. Further studies are needed to assess potential ecotoxicological effects and ensure the safe and sustainable application of nanotechnologies in agriculture and the environment.

In the European Union, current guidelines for the use of nanoparticles in the agri-food sector are outlined by the Scientific Committee Guidance on Nano Risk Assessment by the EFSA [113]. The risk assessment process consists of several phases, including the characterization of materials and of the toxicological hazards. The first phase involves a clear and precise physico-chemical characterization of the nanomaterials, followed by an oral exposure assessment. If the material does not fully dissolve in the food matrix, an additional evaluation phase is required. The final phase includes the identification of toxicological hazards through both in vitro and in vivo testing [113].

In the United States, the use of nanoparticles is regulated by multiple authorities. In the agricultural sector, the main institutions involved are the National Nanotechnology Initiative (NNI), the Environmental Protection Agency (EPA) and the Food and Drug Administration (FDA), as detailed in the National Nanotechnology Initiative Environmental, Health, and Safety Research Strategy: 2024 Update. The NNI provides general guidelines, including exposure limits and voluntary standards. The EPA enforces the Toxic Substances Control Act, while the FDA focuses on drugs and biologic products. Additionally, the Consumer Product Safety Commission (CPSC) and the Occupational Safety and Health Administration (OSHA) are responsible for assessing the impact of nanomaterials on consumer health and establishing exposure limits in the occupational settings [114].

The fate, transport, and bioaccumulation of nanoparticles in soil, plants, and aquatic systems need to be carefully studied [115]. There is also a need to understand the long-term effects of nanoparticle exposure on beneficial soil microorganisms and the food chain [32]. To address these concerns, researchers are focusing on the development of biodegradable and eco-friendly nanoparticles. Regulatory frameworks and safety guidelines are also being established to ensure the responsible use of nanotechnology in agriculture. Transparent risk assessments and stakeholder engagement will be essential to promote public trust and acceptance [32]. The integration of nanoparticles into agricultural practices holds significant potential to advance sustainability goals by improving nutrient use efficiency, reducing agrochemical inputs, enhancing stress tolerance, and preserving environmental resources. However, the success of nano-enabled agriculture depends not only on technological innovation but also on responsible deployment, comprehensive risk assessment, and supportive policy development [116]. Continued interdisciplinary research and collaboration between scientists, farmers, industry, and policymakers will be key to harnessing the full benefits of nanotechnology in shaping the future of sustainable agriculture [117]. However, in the agricultural field, the applications of nanoparticles are still in the early stages of development, with considerable uncertainty regarding their technical and commercial feasibility. This uncertainty consequently limits the adoption of NP for large-scale applications.

While the technology may perform well in the laboratory, scaling up presents challenges related to consistency, reproducibility, and costs, as producing nanomaterials while maintaining the same quality and characteristics achievable in small batches is difficult [118]. Successfully addressing these challenges will be key to making nanofertilizers both affordable and accessible to farmers worldwide, particularly in developing countries where sustainable agriculture and food security are pressing concerns. Since farmers tend to be conservative, they are unlikely to adopt new technologies unless clear benefits are demonstrated, making trust and perceived value crucial factors for the acceptance of nanotechnologies in agriculture [9].

Therefore, it is imperative to validate the numerous promising findings from laboratory-based NP studies highlighted in this review through extensive long-term field-scale assessments. Moreover, uncertainties related to the health and environmental effects of large-scale NP supply, coupled with an insufficient detailed understanding of their impacts in different conditions, must be addressed through a combination of additional laboratory investigations and field-scale pilot projects. In this context, in the future comparative efficiency of different nano-fertilizers, or a SWOT analysis of nanotechnology in agriculture will have to be carried out. In fact, future research should include a comparative assessment of the efficiency of different nano-fertilizers, a SWOT analysis of nanotechnology applications in agriculture, and a thorough evaluation of their scalability and socioeconomic implications. It will also be important to critically examine conflicting evidence regarding their long-term effectiveness and environmental impact.

Adoption barriers—including production costs, smallholder access, consumer acceptance, and ethical considerations—are crucial to real-world implementation and should be addressed in future work.

In summary, nanotechnology offers new ways to solve problems caused by traditional pesticides, which can damage health and environment [95]. It helps create new types of pesticides and delivery methods [119] using tiny particles that can fight many plant diseases and pests, and also help plants absorb nutrients and fight illness [120,121]. Most nanopesticides are still being developed, so more research is needed to check how well they work and if they are safe for the environment [6,103]. Besides pest control, nanotechnology could also help clean pollution and improve soil, but we still need to study its long-term effects on nature [120]. Future work should focus on lowering costs and making these technologies available to everyone to support their use worldwide [122]. Ultimately, nanotechnology holds the potential to revolutionize modern agriculture and contribute significantly to global food security and environmental sustainability.

## Figures and Tables

**Table 1:** Main Nanotechnology Approaches in Sustainable Agriculture.

Nanotechnology Approach	Key Features	Applications	References
Nanoparticles	- High specific surface area - Controlled and sustained release - Responsiveness to stimuli	- Delivery of fertilizers, pesticides, micronutrients, agrochemicals, bioactives (e.g., salicylic acid) - Plant growth regulators - Induced plant resistance - Reduced phytotoxicity - Enhanced germination and growth - Gene delivery	[16,19,20,21]
Nano-formulated agrochemicals (herbicides, insecticides, fungicides, plant growth regulators)	- Improved stability - Solubility - Targeted/stimuli-responsive delivery	- Enhanced weed/insect/disease control - Improved crop performance and safety - Reduced dosage and environmental impact	[15,17,18,22,23]
Nano-fertilizers	- Controlled and sustained release of nutrients	- Improved nutrient-use efficiency - Lower losses and pollution	[24,25]
Nanomaterials for soil and water improvement (nano-clays, TiO_2_ NPs, Fe_3_O_4_)	- Improved soil structure - Water retention - Water purification	- Growth support under stress (e.g., heat, drought) - Slow-release formulations - Removal of heavy metals/pesticides	[26,27,28]
Nano-enabled sensors	- Real-time detection - High sensitivity	- Monitoring soil nutrients and moisture for precision agriculture	[29]
NPs in plant bioengineering	- Enable transient expression and gene studies	- Functional genomics and gene editing in crops	[30]

**Table 2:** Main types of Nanoparticles and their Applications in Agriculture.

Nanotechnology Approach	Composition	Advantages	Applications	Main Outcomes	References
Biopolymeric Nanoparticles	- Natural (e.g., chitosan, alginate) - synthetic polymers (e.g., PLGA, PCL)	- Biocompatibility - Biodegradability - Controlled release - Responsiveness to stimuli - Mucoadhesion, good plant interaction and antifungal properties (for chitosan)	- Carriers of pesticides - Carriers of fertilizers - Plant growth regulators - Delivery of bioactives (e.g., salicylic acid) - Inducement of plant resistance - Reduction in phytotoxicity	Chitosan NPs 1000–5000 ppm: reduced *Fusarium* infection in wheat by 40–60%, with lower phytotoxicity vs. bulk chitosan.	[16,19,20,21]
Carbon-based Nanoparticles	- Carbon nanotubes (CNTs) - Graphene oxide (GO) - Fullerenes - Biochar nanoparticles	- High specific surface area - Strong adsorption capacity - Controlled and sustained release - Enhanced nutrient uptake - Biocompatibility	- Delivery of fertilizers - Pesticides - Micronutrients - Enhanced germination and growth	CNTs applied at 20–100 mg/L increased N, P, K uptake and biomass in maize vs. conventional fertilization.	[39,40,41]
Dendrimers	- Highly branched synthetic polymers (e.g., PAMAM)	- Surface functionalization - High loading capacity - Precise targeting	- Targeted delivery of agrochemicals - Gene delivery	2–3.5, at 1–10 ppb PAMAM with urea + Fe/Mn fertilizer: improved fertilizer mobility and foliar growth.	[42,43]
Mesoporous Silica Nanoparticles (MSNs)	- Inorganic silica with ordered pores	- High specific surface area - Pore size tunability - Controlled release	Controlled delivery of pesticides or nutrients	MSNs 20 nm, 2 nm pores: absorbed by wheat, lupin, *Arabidopsis*; no phytotoxicity; localized in roots, xylem, leaves.	[44,45]
Silica-based NPs (SiO_2_ NPs)		- High specific surface area - Controlled release	- Mitigation of abiotic stress - Improvement of drought resistance/tolerance in crops - Activation of antioxidant enzymes - Regulation of Reactive Oxygen Species (ROS) and Reactive Nitrogen Species (RNS)	SiO_2_ NPs pretreatment (100–800 mg/L): alleviated CuO-NP-induced root inhibition (50% reduction in root growth otherwise); effect reversed in triticale.	[46,47,48]
Metal/Metal Oxide Nanoparticles	- ZnO - TiO_2_ - Fe_2_O_3_ - Fe_3_O_4_ - CuO - Ag	- Improved stress tolerance - Antimicrobial behavior - Nutrient supplementation	- Boosting growth - Disease resistance - Micronutrient delivery - Enhancement of crop growth and yield - Reduction in oxidative/salt stress - Disease control (Ag NPs) - Removal of contaminants (Fe_3_O_4_) - Improvement of water quality - High reactivity; enhance photosynthesis and nutrient uptake; antimicrobial - ROS regulation - Magnetic recovery	Fe_3_O_4_ NPs (200–500 mg/L): enhanced wheat growth, photosynthesis, pigments; increased ascorbate peroxidase, reduced MDA; higher leaf Fe, P, K.	[24,28,49,50,51,52,53,54]
Stimuli-responsive Nanocarriers	- Polymeric nanosystems - Hybrid nanosystems	Release triggered by: pH light enzymes temperature	- Precision delivery under specific plant or soil conditions	Eu@DMSNs/Pec 50–500 mg/L (tomato): enhanced root growth at low doses; no effect on seed germination; antibacterial activity (EC_50_ = 126 mg/L for *B. subtilis*, 165 mg/L for *E. coli*).	[25,55]
Nanoemulsions	- Oil-in-water or water-in-oil emulsions (nano-sized)	- Improved solubility and stability of poorly soluble compounds	- Natural pesticides - Essential oil-based formulations	Caraway oil nanoemulsion up to 10%: safe for maize; caused 50% damage at 5.1% and 90% at 13% in barnyard grass; relative water content ↓ to ~85% (maize) and ~80% (weed).	[56,57]

## Data Availability

Not applicable.
